# The pan-cancer landscape of netrin family reveals potential oncogenic biomarkers

**DOI:** 10.1038/s41598-020-62117-5

**Published:** 2020-03-23

**Authors:** Wenjun Hao, Meng Yu, Jiaxing Lin, Bitian Liu, Haotian Xing, Jieping Yang, Dan Sun, Feilong Chen, Mingzhe Jiang, Chaozhi Tang, Xizhe Zhang, Yongkang Zhao, Yuyan Zhu

**Affiliations:** 1 Department of Urology, The First Affiliated Hospital of China Medical University, Shenyang, 110000 China; 2 Department of Urology, The First Affiliated Hospital of Dalian Medical University, Dalian, 116000 China; 3 Department of Reproductive Biology and Transgenic Animal, China Medical University, Shenyang, 110000 China; 4 Department of Urology, The Second Affiliated Hospital of China Medical University, Shenyang, 110000 China; 5 School of Computer Science and Engineering, Northeastern University, Shenyang, 110000 China; 6 National Institute of Health and Medical Big Data, China Medical University, Shenyang, 110000 China; 7 Joint Laboratory of Artificial Intelligence and Precision Medicine of China Medical University and Northeastern University, Northeastern University, Shenyang, 110000 China

**Keywords:** Cancer genomics, Data mining

## Abstract

Recent cancer studies have found that the netrin family of proteins plays vital roles in the development of some cancers. However, the functions of the many variants of these proteins in cancer remain incompletely understood. In this work, we used the most comprehensive database available, including more than 10000 samples across more than 30 tumor types, to analyze the six members of the netrin family. We performed comprehensive analysis of genetic change and expression of the netrin genes and analyzed epigenetic and pathway relationships, as well as the correlation of expression of these proteins with drug sensitivity. Although the mutation rate of the netrin family is low in pan-cancer, among the tumor patients with netrin mutations, the highest number are Uterine Corpus Endometrial Carcinoma patients, accounting for 13.6% of cases (54 of 397). Interestingly, the highest mutation rate of a netrin family member is 38% for NTNG1 (152 of 397). Netrin proteins may participate in the development of endocrine-related tumors and sex hormone-targeting organ tumors. Additionally, the participation of NTNG1 and NTNG2 in various cancers shows their potential for use as new tumor markers and therapeutic targets. This analysis provides a broad molecular perspective of this protein family and suggests some new directions for the treatment of cancer.

## Introduction

Cytokines, growth factors, angiogenic factors, and extracellular proteases are secreted to the outside of the cell to produce biological functions^1^. These secreted proteins participate in the immune regulation of chronic inflammation^2,3^ and the occurrence of lipid metabolism diseases^4^, but also play important roles in tumor invasion, metastasis, immunity, and drug resistance^5–8^. For example, CD317 and EGFR are secreted proteins that are potential diagnostic markers for non-small cell lung cancer^9^. CD63 is secreted by melanoma into the blood and can be used as a protein marker^10^. HGF inhibits the treatment of RAF inhibitors of BRAF mutant melanoma^11^.

The netrins are neuroguiding factors with axonal guiding function, and are similar to laminin in structure. Netrins were first discovered in *C. elegans* in 1990^12^, and this family of proteins includes the secreted proteins Netrin-1 (NTN1), Netrin-3 (NTN3), Netrin-4 (NTN4), and Netrin-5 (NTN5). The secreted proteins have a common domain structure, with an N-terminal laminin repeat (Laminin N-terminal), three cysteine-rich EGF modules (V-1, V-2, and V-3), and a positively charged C-terminal domain (NTR)^13^. The netrin family also includes two membrane-binding proteins, Netrin-G1 (NTNG1) and Netrin-G2 (NTNG2)^14^. Although these proteins also have Laminin N-terminal and Laminin EGF domains, their ends have different functions due to GPI^15^. The major binding receptors of the secreted netrin proteins are DCC and UNC5 homologue family UNC5A-D, which are both dependent receptors. Netrin binding to a receptor promotes cell survival, proliferation, and differentiation, and without netrin binding, the receptor induces apoptosis^16,17^. Netrins play seemingly contradictory cellular functions through downstream signal transduction cascades, including promotion of tumor cell proliferation, migration, invasion, and angiogenesis, and inhibition of tumor development and angiogenesis^18^. Netrin-1 promotes the invasion and angiogenesis of glioblastoma cells by activating RhoA, cathepsin B, and cAMP response element binding protein or the Notch pathway^19,20^. Inflammation promotes colon cancer development by increasing NTN1 expression^21^. NTN4 promotes the proliferation of glioblastoma cells by activating the AKT-mTOR signaling pathway^22^. Overexpression of Netrin-4 inhibits colorectal tumor progression and angiogenesis through the VEGF/VEGF receptor pathway^23^. NTN1 inhibits the growth of early pancreatic cancer cells by inhibiting the MEK/ERK pathway and ITGB4^24^. However, netrin proteins can have different effects in different types of cancers, and can either inhibit or promote cancer. Thus, it is challenging to predict appropriate treatment interventions based on behavior of one kind of netrin protein in different cancers or and difficult to predict the complex effects of multiple netrins in cancer.

In this study, we comprehensively analyzed the molecular characteristics of all members of the netrin family in pan-cancer. Using a large dataset, we analyzed the potential cancer biological functions and common characteristics of netrin proteins in different aspects of cancer.

## Results

### Mutation and Fusion Gene Analysis of Netrin Family in pan-cancer

We obtained data for 10436 patients with mutation information from the cBioPortal website (www.cbioportal.org/)^25,26^, using the TCGA PanCancerAtlas for Mutual Exclusivity analysis of pan-cancer mutations. We found co-occurrence relationships of NTN3, NTN4, NTN5, NTNG1, and NTNG2 with NTN1; NTN3 with NTN4, NTNG1 and NTNG2; NTN4 with NTNG1 and NTNG2; NTN5 with NTNG1; and NTNG1 with NTNG2. All relationships had significance (p < 0.05), but co-occurrence relationship was not found in any TCGA (The Cancer Genome Atlas) single cancer.

**Figure 1:**
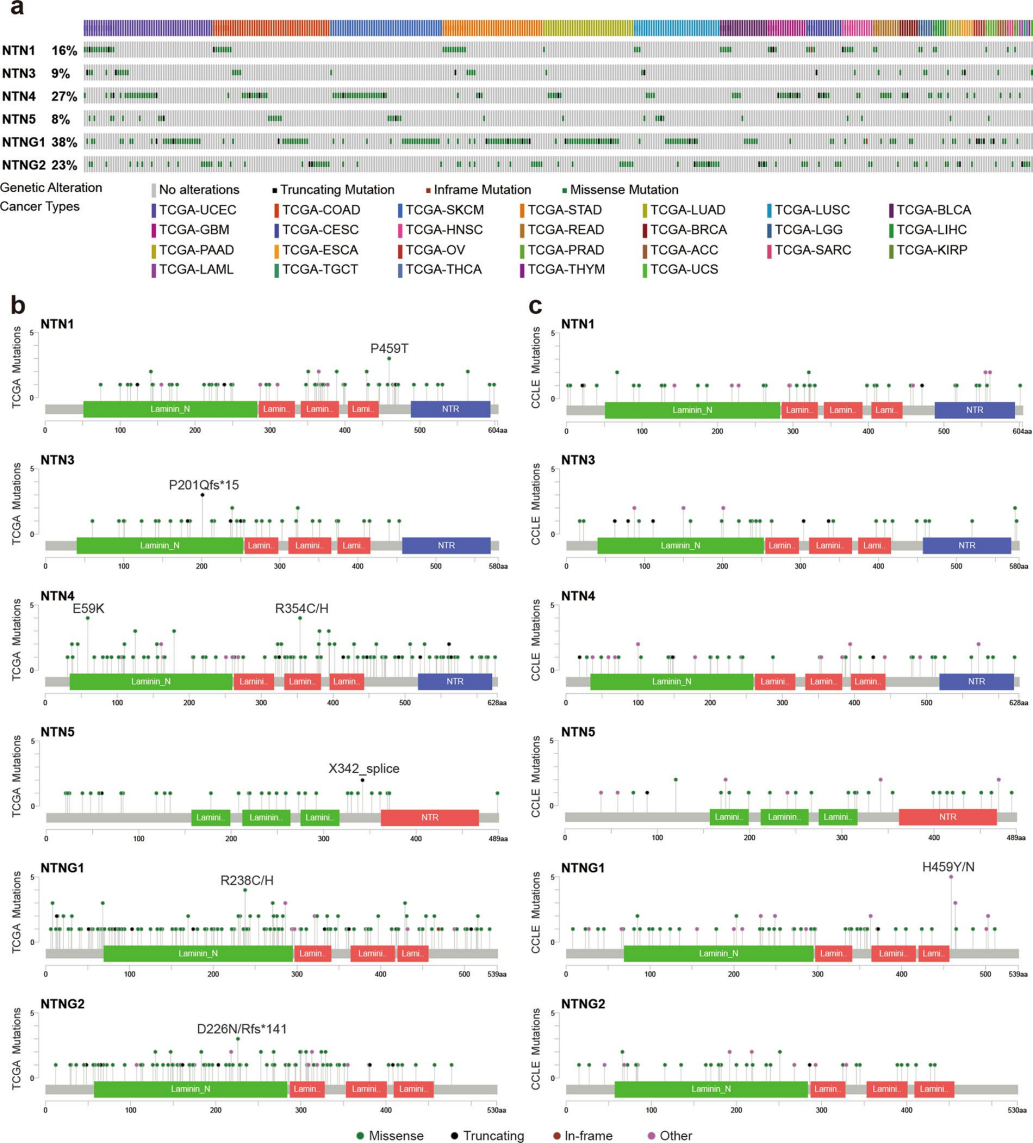
Netrins mutations in cancer. (**a**) Netrins exhibit non-synonymous mutations in the coding region in 26 kinds of TCGA cancers. Each gray vertical bar represents a patient. (**b**) Amino acid mutation of netrins in TCGA cancers. Hot spot mutations are indicated. Substituted mutations are represented by slash-separated single-letter amino acid codes. Other indicates important mutation sites that predicted as damaging in both VEST3 and REVEL algorithms in the VEST3 and REVEL algorithms and coverage < 0.1 in the functional and structural importance of the protein sequence position. Fs, frameshifts; *, termination codon. (**c**) Amino acid mutation of netrins in Cancer Cell Line Encyclopedia (CCLE). Substituted mutations are represented by slash-separated single-letter amino acid codes. Other represents a mutation site consistent with TCGA mutation in CCLE.

Mutations in the netrins were identified in the 33 cancers included in TCGA (Fig. 1a). At the cancer level, netrins associated with uterine corpus endometrial carcinoma (UCEC) exhibited the highest number of mutations (54), followed by colon adenocarcinoma (COAD) (49), skin cutaneous melanoma (SKCM) (47), stomach adenocarcinoma (STAD) (42), lung adenocarcinoma (LUAD) (38), and lung squamous cell carcinoma (LUSC)(36). The total mutation rates of Netrin family members in the above six cancers were 10.19%, 12.28%, 10.06%, 9.61%, 6.70% and 7.32%, respectively. In kidney renal papillary cell carcinoma (KIRP) and thyroid carcinoma (THCA), only two and one patient mutations in netrins were identified, respectively. Analysis revealed that the different genes contained hot spots of mutations (Fig. 1b). Seven hot spot mutations were not predicted to be damaging according to both the VEST3 and REVEL algorithms, with coverage <0.1 for the functional and structural importance of the protein sequence. However, the hot spot mutation P201Qfs*15 of NTN3 was identified in three patients, each with a different cancer (ESCA, STAD, UCEC), and encodes a truncated protein. E59K, one of the hotspot mutations of NTN4, was detected in four patients with three cancers (READ, COAD, and UCEC) and both VEST3 and REVEL algorithms indicated this change was dual-damaging. The hot spot mutation X342_splice of NTN5 occurred in two cancers (SKCM and UCEC) in two patients, and also encodes a truncated protein. The hot spot mutation R238C/H of NTNG1 was detected in three patients with three cancers (COAD, STAD, and UCEC), and R238C was predicted as damaging in VEST3 and REVEL algorithms. The NTNG2 hotspot mutation D226N/Rfs*141 occurred in three patients with three cancers (LAML, GBM, and COAD), and NTN1 hotspot mutation P459T was detected in three patients with two cancers (STAD, READ).The mutations in NTN1, NTN3, NTN4, NTNG1, and NTNG2 were mainly in the laminin-N domain, and most mutations of NTN5 were concentrated in the laminin_EGF2 domain. The mutation distribution of NTN family members of CCLE showed most mutations were concentrated in the laminin-N domain (Fig. 1c), and most multiple mutations were also found in TCGA.

**Figure 2:**
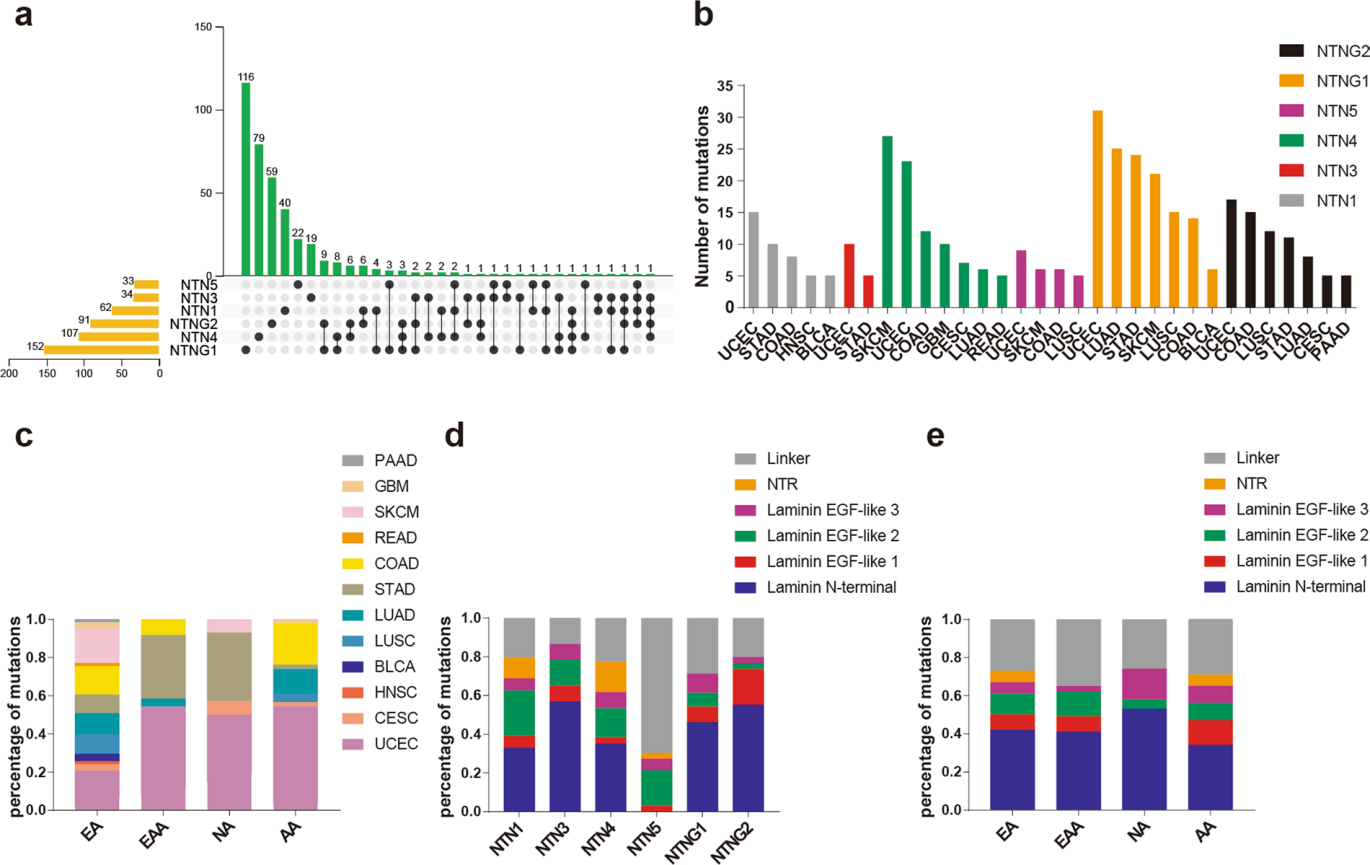
Distribution of netrin mutations in individuals and proteins. (**a**) The intersection of mutations in individuals of members of the netrin family. (**b**) The number of mutations in the netrin family in TCGA cancers. (**c**) Percentage map of the distribution of cancers associated with mutations in netrin genes (number of mutations ≥ 5) among genetic ancestry groups. (**d**) Percentage distribution of mutations in each member of the netrin family in its domain. (**e**) Percentage map of mutations in the netrin family domain among genetic ancestry groups.

After screening by genetic ancestry groups and non-synonymous mutations, 62 patients had complete mutation information (64 mutations) for NTN1, 34 patients were included with complete mutation information (37 mutations) for NTN3, and 107 patients had complete mutation information (120 mutations) for NTN4. Additionally, the data included 33 patients with 33 mutations in NTN5, 152 patient and 161 mutations in NTNG1, and 91 patients with 98 mutations in (Fig. 2a, Supplementary Data 1). By comparing the data, we found that up to four netrin family members in individual patients contained mutations in four patients, three UCEC patients and one STAD patient with mutations in both NTN1 and NTNG2. In cancer studies with at least five mutations in members of netrin genes (Fig. 2b), UCEC patients were one of the two highest among the cancers associated with the netrin family mutations, with most mutations in NTNG1. SKCM has the most mutations in NTN4. From the perspective of ethnic distribution (Fig. 2c), the highest number of cancer mutations among the four genetic ancestry groups was UCEC, and the netrin family mutations in STAD were mainly distributed in EAA and NA. Most netrin mutations in COAD occur in EA and AA. Further analysis of the mutations (Fig. 2d,e) revealed that most mutations in netrins were in the laminin N-terminal domain, except for NTN5. In the laminin EGF domains of NTN1, NTN3, NTN4, and NTN5, most mutations were in the laminin EGF-like 2 domain. In the NTNG1 laminin EGF domains, the mutation of laminin EGF-like 3 was the most frequent, and in the NTNG2 laminin EGF domains, the mutation of laminin EGF-like 1 was the most frequent. The mutations in the NTR domain were mainly concentrated in NTN1 and 4. Overall, mutations in netrin genes in each genetic ancestry group were mainly concentrated in the laminin N-terminal and laminin EGF domains. Based on the distribution of the mutations in genetic ancestry groups, we found that most netrin mutations occurred in UCEC, and the terminal and laminin EGF domains were the main mutation areas.

**Figure 3:**
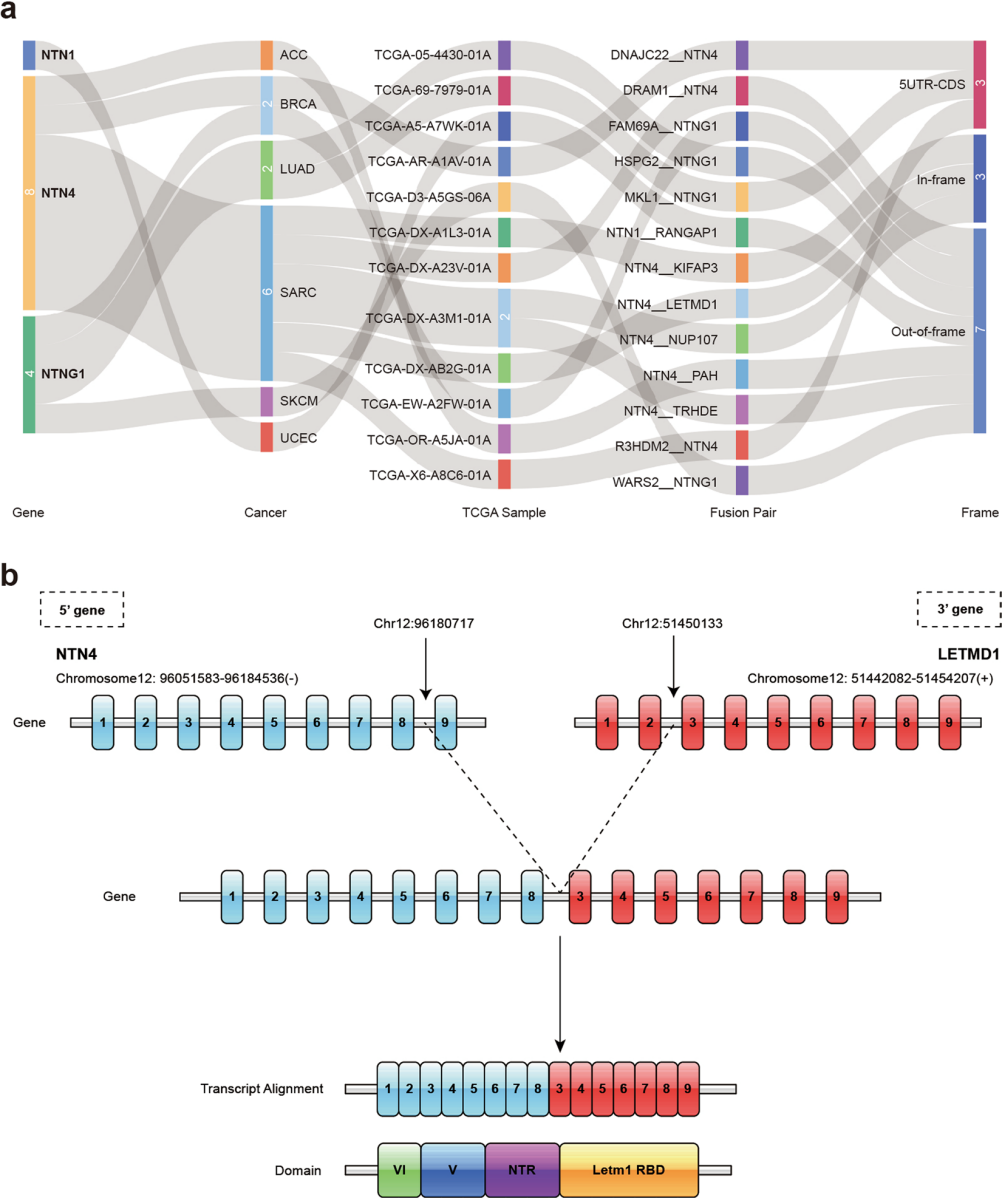
Fusion gene of netrins. (**a**) Fusion gene of NTN1, NTN4, NTNG1 in cancer. (**b**) NTN4 and LETMD1 fusion transcription. Blue indicates the exon of the NTN4 gene, red indicates the exon of the LETMD1 gene, and the dotted line indicates the linkage of the two partial genes.

**Figure 4:**
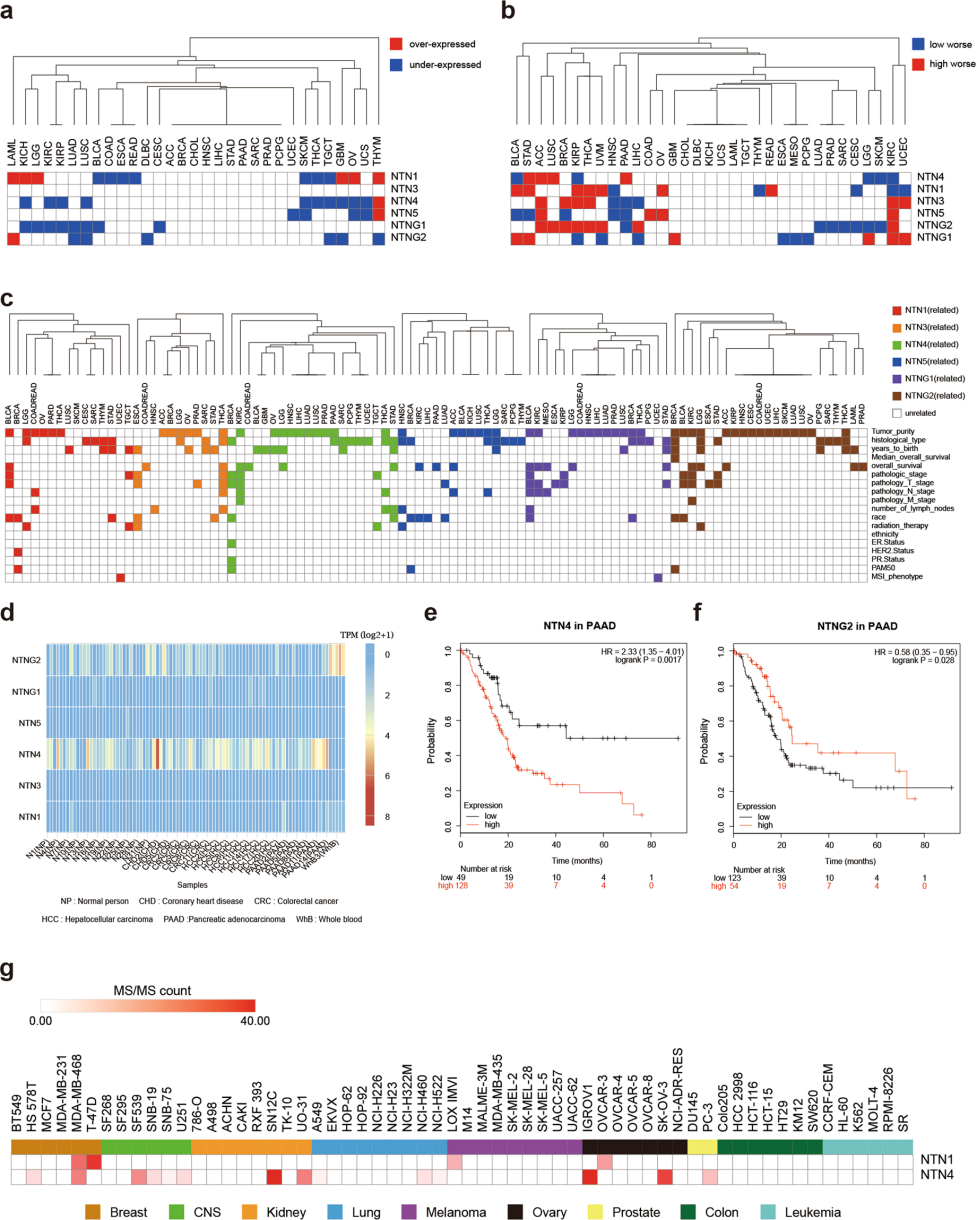
Netrin expression and clinical features. (**a**) Differential expression of netrins between TCGA cancers and their normal tissues. Blue indicates low expression in cancer tissue (p < 0.05), red indicates high expression in cancer tissue (p < 0.05). (**b**) Differential survival between high and low expression of netrins in TCGA cancers. Blue indicates worse survival of low expression in cancer tissue (p < 0.05), and red indicates worse survival of high expression in cancer tissue (p < 0.05). (**c**) The relationship between netrin expression and the clinical parameters of TCGA cancer. Color indicates that clinical parameters were associated with gene expression (p < 0.05), and white indicates the opposite. (**d**) Heat map of netrin expression in human blood. (**e**) The survival curve of NTN4 in pancreatic cancer (optimal cut off). (**f**) The survival curve of NTNG2 in pancreatic cancer (optimal cut off). (**g**) Expression heatmap of NTN1 and NTN4 as extracellular vesicle proteins in 60 cell lines of NCI-60.

In tumorigenesis, fusion genes produced by genomic level cleavage and resplicing are often targets for tumor diagnostic treatment. We detected fusion transcripts of the NTN family from 33 TCGA cancers with high confidence by using the TCGA Fusion Gene Database^27^ (Fig. 3a, Supplementary Data 2). The NTN1 fusion gene in UCEC is NTN1__RANGAP1. NTNG1 fusion transcripts were detected in BRCA (1), LUAD (2), and SKCM (1), and NTN4 fusion transcripts were detected in ACC (1), BRCA (1), and SARC (6). LETMD1 (Human Cervical Cancer Oncogene) was detected fused to NTN4 in Sarcoma (SARC) (Fig. 3b). Expression of both NTN4 and LETMD1 is related to the SARC histological_type (Fig. 4c). The NTN4__LETMD1 protein combines the effective functional domains of both proteins, which may contribute to SARC. Similarly, two fusion transcripts were found in LUAD, including MKL1__NTNG1. MKL1 is a transcriptional cofactor, and was found fused to the RBM15 gene in acute megakaryoblastic leukemia^28^. Additionally, MLK1 enhanced cancer cell migration and invasion by epigenetic activation of MMP9 transcription in lung cancer^29^. Thus, MKL1__NTNG1 may be a target for the treatment of lung adenocarcinoma.

### Expression and clinical analysis of netrins in pan-cancer

We performed differential expression analysis of 33 TCGA cancers by Gene Expression Profiling Interactive Analysis (GEPIA)^30^ (Fig. 4a, Supplementary Data 3). We identified significant differential expression between cancer tissue and paracancerous tissue (p < 0.05). There was differential expression of NTN1 and NTN4 in endocrine hormone-related cancers THCA, OV, and THYM. NTNG1 and NTNG2 were differentially expressed in LUAD and LUSC. NTN1 was differentially expressed in nerve-related cancers: LGG and GBM. NTNG1 was differentially expressed in pan-kidney: KIRP and KIRC. There was no differential expression of NTN3.

We analyzed the survival curves of 33 kinds of TCGA cancers by Kaplan-Meier Plotter^31^ and GEPIA (Fig. 4b, Supplementary Data 4) and found that netrins had no effect on the survival of CHOL, DLBC, KICH, UCS, LAML, and TGCT. In pan-kidney (KIRP and KIRC), almost all members of the netrin family affect survival. The high expression of NTNG1 in LUSC and the low expression of NTNG1 in LUAD correlated with worse survival. NTN4 and NTNG1 are involved in the survival of most urinary tumors (BLCA, ACC, KIRC, KIRP, and PRAD). In endocrine hormone-related cancers (ACC, THCA, PAAD, OV, and THYM), the expression of NTN1 was related to the survival of THCA, THYM, and OV, and the expression of NTN3 was related to the survival of ACC, THCA, and PAAD. The expression of NTN5 was related to the survival of ACC, PAAD, and OV.

We carried out a comprehensive expression-clinical analysis of all members of the netrin family in 32 types of TCGA cancer through LinkedOmics(Fig. 4c, Supplementary Data 5). In pan-kidney (KIRP and KIRC), NTNG1 was related to pathology T stage of KIRP and KIRC, and NTN4 was related to pathology T stage, pathology N stage, and pathology M stage of KIRC. In BRCA, the expression of NTN1, NTN4, NTN5, and NTNG2 was related to its PAM50 typing, and the expression of NTN1 was related to its HER2 status and the expression of NTN4 was related to its ER status. The expression of NTN1 and NTNG1 was related to the MSI phenotype of UCEC. The expression of NTN3 and NTN4 was related to pathology N stage and number of lymph nodes of THCA. The expression of NTN1, NTNG1, and NTNG2 was related to pathologic stage, pathology T stage and race of BLCA, and NTNG1 was related to pathology N stage and number of lymph nodes of BLCA. The expression of NTN1 and NTN4 was related to radiation therapy and pathologic stage in Testicular Germ Cell Tumors (TGCT).

The expression data of NTN family members in normal (NP), coronary heart disease (CHD), colorectal cancer (CRC), hepatocellular carcinoma (HCC), pancreatic cancer (PAAD) and breast cancer (BC) was obtained from exoRBase^32^ (Fig. 4d). NTN1, NTN3, NTN5, and NTNG1 were expressed in low abundance in blood. In contrast, the expression of NTN4 and NTNG2 in blood was relatively high. The expression of NTN4 was high in the blood of PAAD (up), and expression of NTNG2 was low in the blood of PAAD (down). Analysis of the optimal survival curve (Fig. 4e) revealed worse survival of high expression of NTN4 in PAAD, and worse survival for low expression of NTNG2. The expression trends of NTN4 and NTNG2 in PAAD tissues and in its blood were consistent.

Examining data for 60 cell lines at the National Cancer Institute (NCI-60)^33^ (Fig. 4g, Supplementary Data 6), we found that NTN1 protein is present in extracellular vesicles in breast, melanoma, and ovarian cancer. NTN4 protein appears as extracellular vesicles in breast cancer, brain tumor, kidney cancer, lung cancer, ovarian cancer, and prostate cancer, and also in human urine^34^. In addition, previous work detected mRNA for NTN1 and NTNG2 in the extracellular matrix of colon cancer^35^.

### Methylation and clinical analysis of netrins in pan-cancer

**Figure 5:**
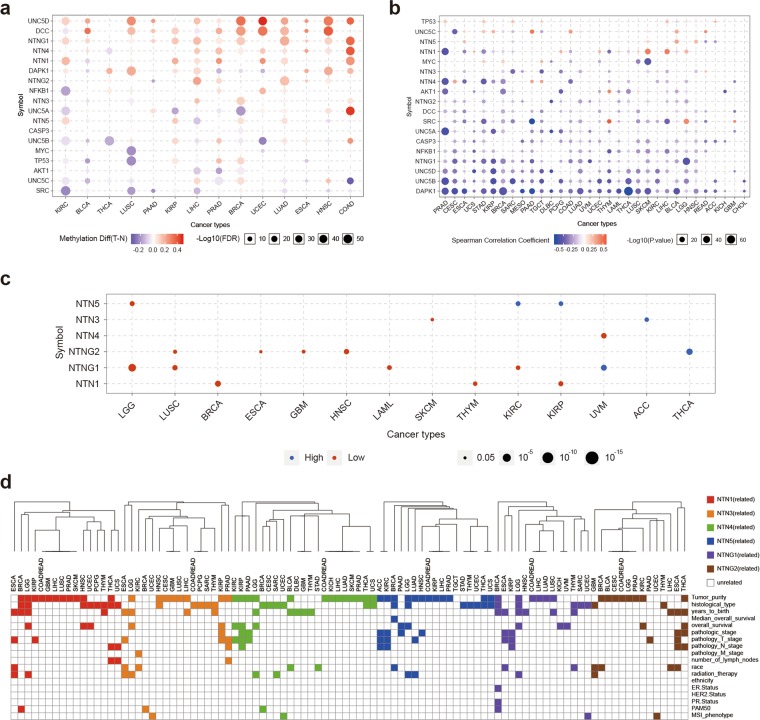
Netrins methylation and distribution in clinical samples. (**a**) Bubble map of the differential methylation of netrins and downstream genes between TCGA cancer and normal samples. Blue dots represent methylation down-regulation in tumors, and red dots represent methylation up-regulation in tumors, with the darker the color, the greater the difference. The size of the point represents statistical significance, where the larger the size, the greater the significance. (**b**) Correlation between methylation and netrin expression and downstream genes in TCGA cancer. Blue dots indicates gene methylation level is up-regulated and gene expression is down-regulated. Red dots indicate that gene methylation level and gene expression are up-regulated. The darker the color, the higher the correlation. The size of the point represents the statistical significance, where the larger the size, the greater the significance. (**c**) Differential survival of high and low methylation of netrins in TCGA cancer. Only genes with p value < 0.05 are displayed on the diagram. Red dots represent worse survival of hypermethylation in tumors, and blue dots indicate the opposite. The size of the point represents the statistical significance, where the larger the size, the greater the significance. (**d**) Relationship between netrin methylation and clinical parameters in TCGA cancers. Color indicates that clinical parameters are associated with methylation (p < 0.05), and white indicates no association.

We analyzed the methylation of the NTN family members and their downstream genes in TCGA 33 cancers using GSCALite^36^ (Fig. 5a–c). Previous studies found that Netrin-1 and its downstream genes are co-methylated in breast cancer^37,38^. Here, we found up-regulated methylation of NTN3, NTN4, NTN5, and NTNG1 and downstream genes UNC5C, UNC5D, DCC, and TP53 in breast cancer. Similar co-methylation patterns of netrins and downstream genes are also present in cancers such as UCEC, LUAD, ESCA, HNSC, and COAD (Fig. 5a). In addition, the expression of members of the netrin family was mainly negatively correlated with methylation, with only a few positive correlations (Fig. 5b). The relationship between methylation and survival (Fig. 5c) showed the survival was worse for hypomethylation of NTN1 in kidney renal papillary cell carcinoma (KIRP) and worse for NTNG1 in kidney renal clear cell carcinoma (KIRC), which is consistent with the correlation of high expression of NTN1 in KIRP and NTNG1 in KIRC with worse survival. This may indicate that the carcinogenic mechanism of NTN1 and NTNG1 in pan-kidney cancers is related to promoter methylation.

We performed a comprehensive methylation-clinical analysis of all members of the NTN family in 32 TCGA cancers using LinkedOmics^39^ (Fig. 5d, Supplementary Data 5). Methylation of NTN1, NTN3, NTN4, NTN5, and NTNG1 was associated with radiation therapy and overall survival of LGG. The methylation of NTNG2 was related to radiation therapy of GBM. In pan-kidney (KIRP and KIRC), methylation of NTN4 was associated with pathology T stage and overall survival of KIRP and KIRC. Methylation of NTN1 and NTNG1 was associated with pathology T stage and pathology N stage of KIRP. NTN1 was associated with pathology N stage and number of lymph nodes in THCA. Methylation of NTN1 and NTN3 was associated with radiation therapy and pathology T stage of ESCA. Methylation of NTN3 was associated with pathology T stage, pathology N stage and number of lymph nodes of PRAD. Methylation of NTN1, NTN3, NTN4, and NTNG1 was associated with PAM50 typing of BRCA, and NTNG1 was also associated with ER.Status and PR.Status. In UCEC, methylation of NTN3, NTN4, NTNG1, and NTNG2 was associated with MSI phenotype.

### Transcription and epigenetics analysis of netrin family genes in pan-cancer

**Figure 6:**
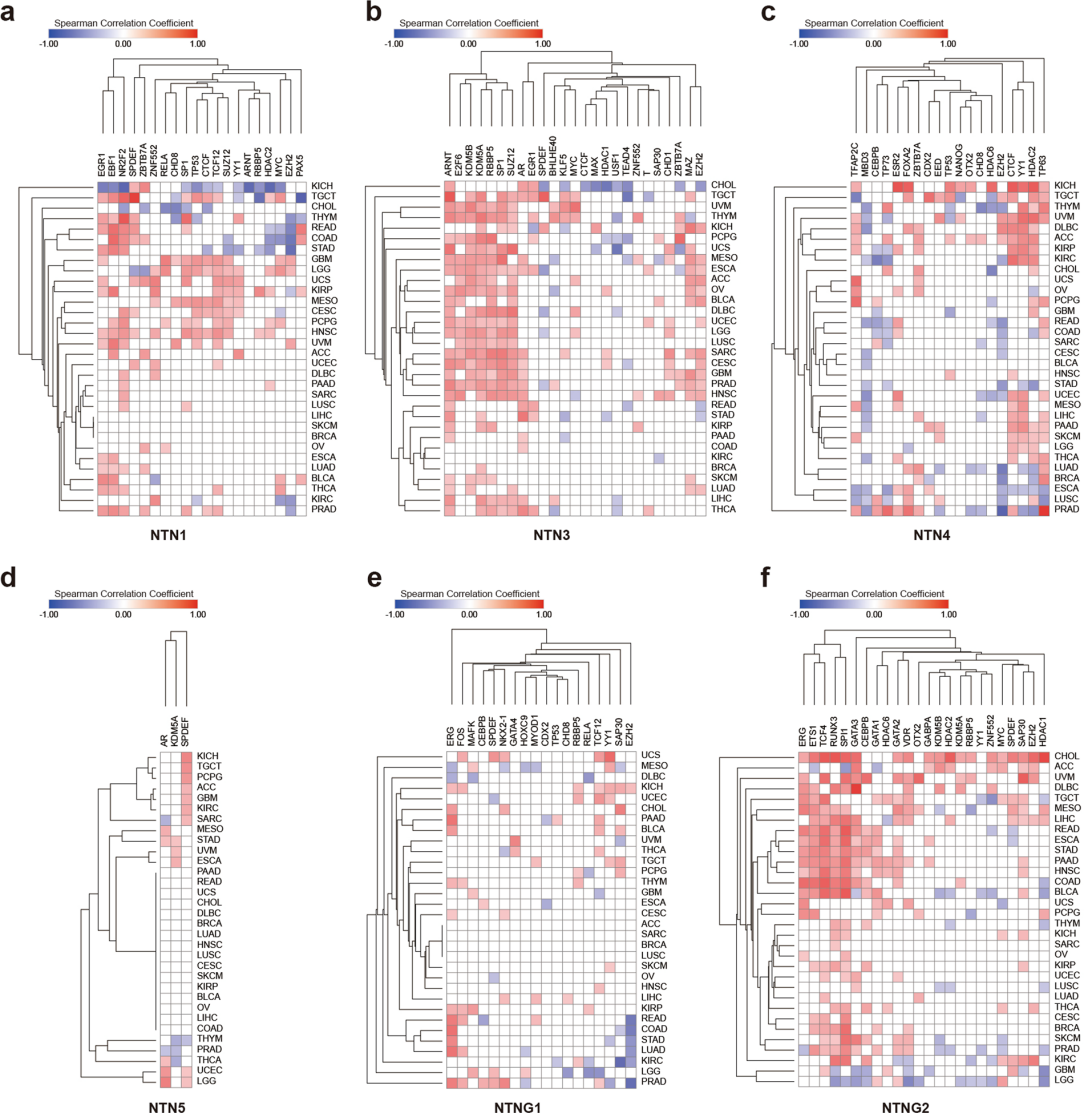
Co-expression of netrin transcription regulators and netrins in 32 kinds of TCGA cancer. Co-expression of transcription regulators of (**a**) NTN1, (**b**) NTN3, (**c**) NTN4, (**d**) NTN5, (**e**) NTNG1, and (**f**) NTNG2 in 32 kinds of TCGA cancers. We used ±0.2 as the cut off value, -0.2 < Spearman Correlation Cofficient < 0.2 as irrelevant. Red indicates a positive correlation to co-expression between the gene and its transcriptional regulator in cancer, while blue is opposite. The darker the color, the higher the correlation.

We downloaded the co-expression data for netrin family members and transcription factors with binding sites located within the 1 kb upstream or downstream of the promoter, as well as chromatin remodeling factors in TCGA 32 cancers from ChIPBase v2.0^40^ (20 NTN1 modifiers, 25 NTN3 modifiers, 19 NTN4 modifiers, 3 NTN5 modifiers, 18 NTNG1 modifiers, and 24 NTNG2 modifiers) (Fig. 6, Supplementary Data 7). AR (androgen receptor) is a transcription factor associated with the development of prostate cancer. When AR is up-regulated, it promotes the development of prostate cancer. Overexpression of EZH2 is also associated with the development of prostate cancer, and phosphorylated EZH2 can act as a co-activator of transcription factors, such as promoting the expression of AR^41,42^. Here, we found that NTN3 transcription is positively regulated by both EZH2 and AR in prostate cancer (r = 0.2297 and 0.2863, respectively)(Fig. 6b), suggesting that NTN3 may play a oncogenic role in prostate cancer. EZH2 may be used as a new diagnostic marker to distinguish thymic squamous cell carcinoma from type B3 thymoma^43^. The transcription of NTN1 in THYM is negatively regulated by EZH2 (r = −0.4056)(Fig. 6a), suggesting that the low expression of NTN1 in thymoma is related to the occurrence and development of THYM. The up-regulation of transcription factor ETS1 is related to the invasion of breast cancer^44^. The transcription of NTNG2 in breast cancer is positively regulated by ETS1 (r = 0.2971)(Fig. 6f), suggesting that NTNG2 may be related to the development of breast cancer. Studies have shown that thyroid cancer promotes the growth and invasion of cancer cells through the mTOR pathway and MYC^45^. The transcription of NTN1 and NTNG2 is positively regulated by MYC in THCA, suggesting that NTN1 and NTNG2 may also participate in the development of thyroid carcinoma.

**Figure 7:**
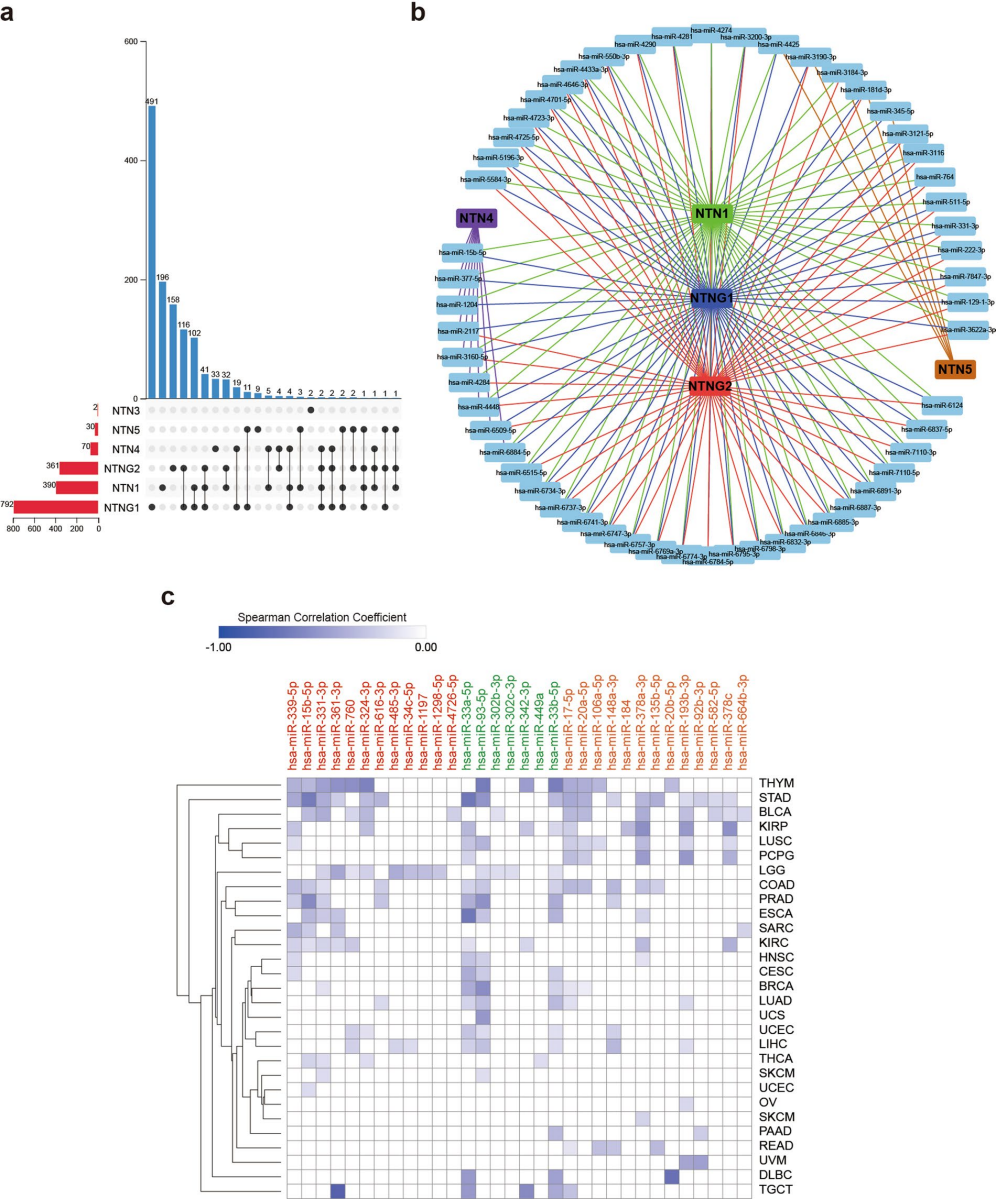
Identification of mirRNA that may target netrins. (**a**) Statistical distribution of mirRNA and Netrins. (**b**) MirRNA that are associated with at least three members of the netrin family. (**c**) The expression correlation heat map between 32 mirRNA and netrin family members in pan-cancer. Red mirRNAs have the potential to bind to NTN1, green mirRNAs have the potential to bind to NTN4, and orange mirRNAs have the potential to bind to NTNG1. The darker the color, the higher the correlation.

Using miRWalk^46^ (including miRBase, TargetScan, miRDB, and miRTarBase, using the TarPmiR algorithm), we predicted a total of 1238 mirRNA that could potentially target the 3′UTR of netrin transcripts (Fig. 7a, Supplementary Data 8). In terms of the total number of mirRNA that could potentially target a gene or the total number of mirRNA that could potentially target a single gene, the highest number of mirRNA could target NTNG1, followed by those targeting NTN1 and NTNG2. Netrin family members mostly bound to a specific mirRNA, but 28.2% of mirRNA could bind to multiple netrins. Statistical analysis indicated that 55 mirRNA were associated with at least three members of the netrin family (Fig. 7b). Comparing these sequences with the data from STARBASE v3.0^47^ (Supplementary Data 9), 42 mirRNA were included in both databases. We further used FDR < 0.05 and negative correlation as screening criteria to observe the expression correlation between mirRNA and the netrin family members for 32 kinds of TCGA cancers (Fig. 7c). We found that half of the mirRNA in thymic carcinoma had a strong inhibitory effect on NTN1. Among the many statistically significant results, hsa-miR-361-3p had the strongest inhibitory effect on NTN1 in TCGT (r = 0.612), hsa-miR-33a-5p had the strongest inhibitory effect on NTN4 in TCGT and ESCA (r = −0.494), and hsa-miR-20b-5p had the strongest inhibitory effect on NTNG1 in DLBC (r = −0.528).

### Pancan-eQTL analysis of netrins in pan-cancer

**Figure 8:**
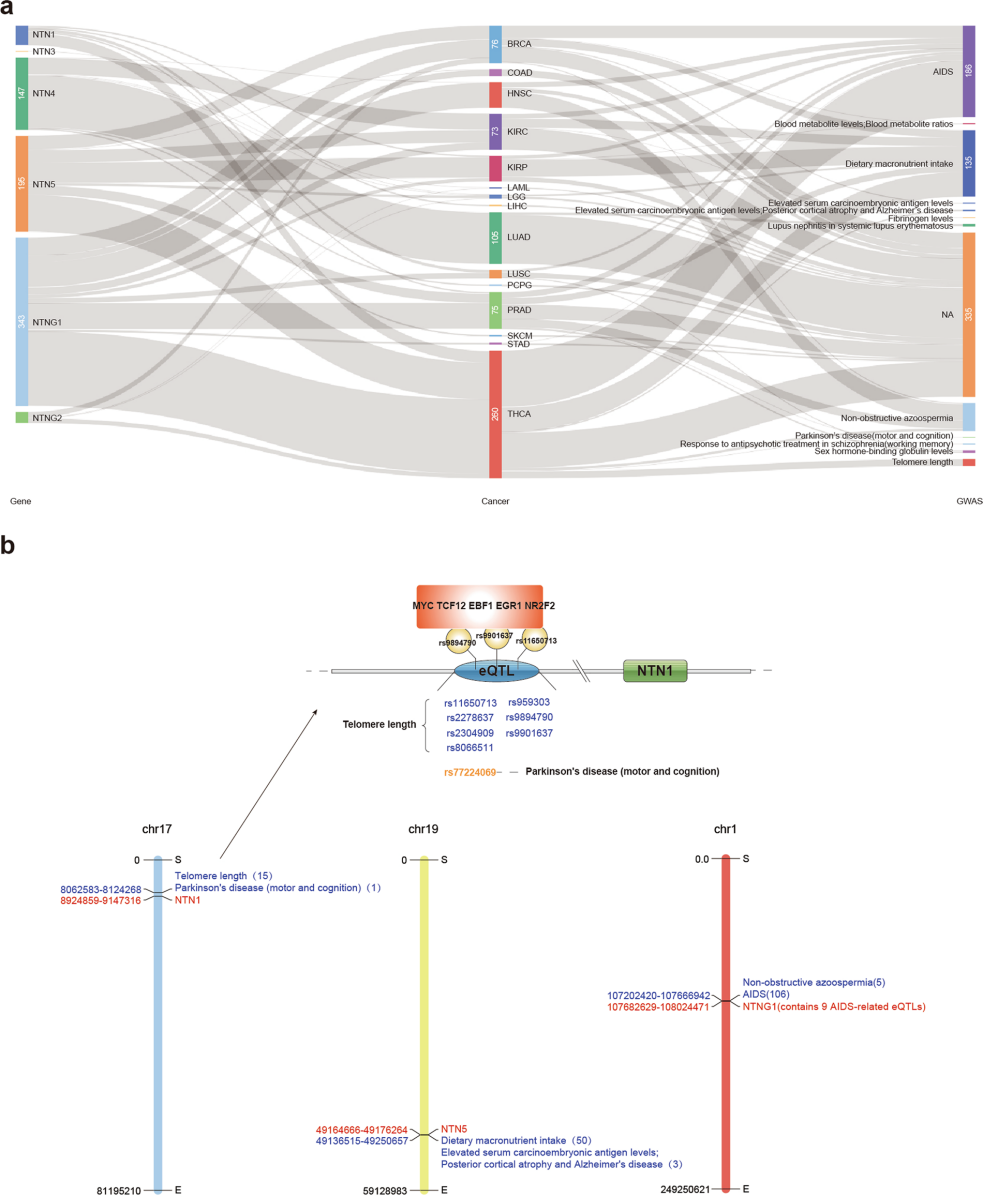
Netrin Cis-eQTLs in TCGA. (**a**) Distribution of netrin Cis-eQTLs in TCGA cancers and corresponding GWAS-related traits. NA indicates that it is not correlated with GWAS traits. (**b**) The distribution of NTN1, NTN5, and NTNG1 Cis-eQTLs and the corresponding GWAS-related traits in THCA and the possible mechanism of NTN1 eQTL in THCA.

We searched for Cis-eQTLs (SNPs within 1 Mb of the gene transcriptional start site) associated with netrin family members in the TCGA 33 cancers by PancanQTL^48^. Most eQTLs identified in cancer samples are cancer-specific, so we can determine the relationships between netrin eQTLs in cancer and complex diseases after correlating with the GWAS (Genome-wide association study) data (Fig. 8a, Supplementary Data 10). We found that several eQTL in KIRP, LIHC, LUSC, PRAD, and THCA affected the expression of NTN1, including 15 eQTLs in THCA that could be associated with GWAS. One eQTL in LGG affected NTN3 expression. Additionally, eQTLs affect the expression of NTN4 in HNSC, KIRC, LAML, LGG, LUAD, PCPG, SKCM, and other cancers, including 5 eQTL that are associated with GWAS in LUAD. The expression of NTN5 was affected by eQTL in BRCA, COAD, KIRC, KIRP, LGG, PRAD, and THCA. Up to 143 NTN5 eQTL were associated with GWAS, of which 135 (94.4%) of the eQTLs were associated with dietary macronutrient intake. A total of 343 eQTLs in BRCA, COAD, HNSC, KIRC, KIRP, LUSC, PRAD, STAD, and THCA affected the expression of NTNG1, and 249 eQTLs were associated with GWAS in these cancers (other than COAD and STAD). Of these, 186 (74.4%) NTNG1 eQTLs were associated with AIDS and 57 (22.9%) were associated with non-obstructive azoospermia. EQTL affects the expression of NTNG2 in BRCA, THCA, and other cancers. We determined that netrin family members have more eQTLs affecting gene expression in THCA and most can be associated with GWAS (Fig. 8b). Seven eQTLs (telomere length) and one eQTL(Parkinson’s disease (motor and cognition)) associated with NTN1 show effects on transcription factor binding. Further, three eQTLs (rs9894790, rs9901637, and rs11650713) were found to affect the binding of MYC, TCF12, EBF1, EGR1, and NR2F2. Additionally, high expression of NTN1 in thyroid carcinoma was correlated with a worse survival. Thus, these eQTLs may alter the activity of these transcription factors to generally promote NTN1 transcription, resulting in thyroid cancer.

### Drug and pathway analysis of Netrin family in pan-cancer

**Figure 9:**
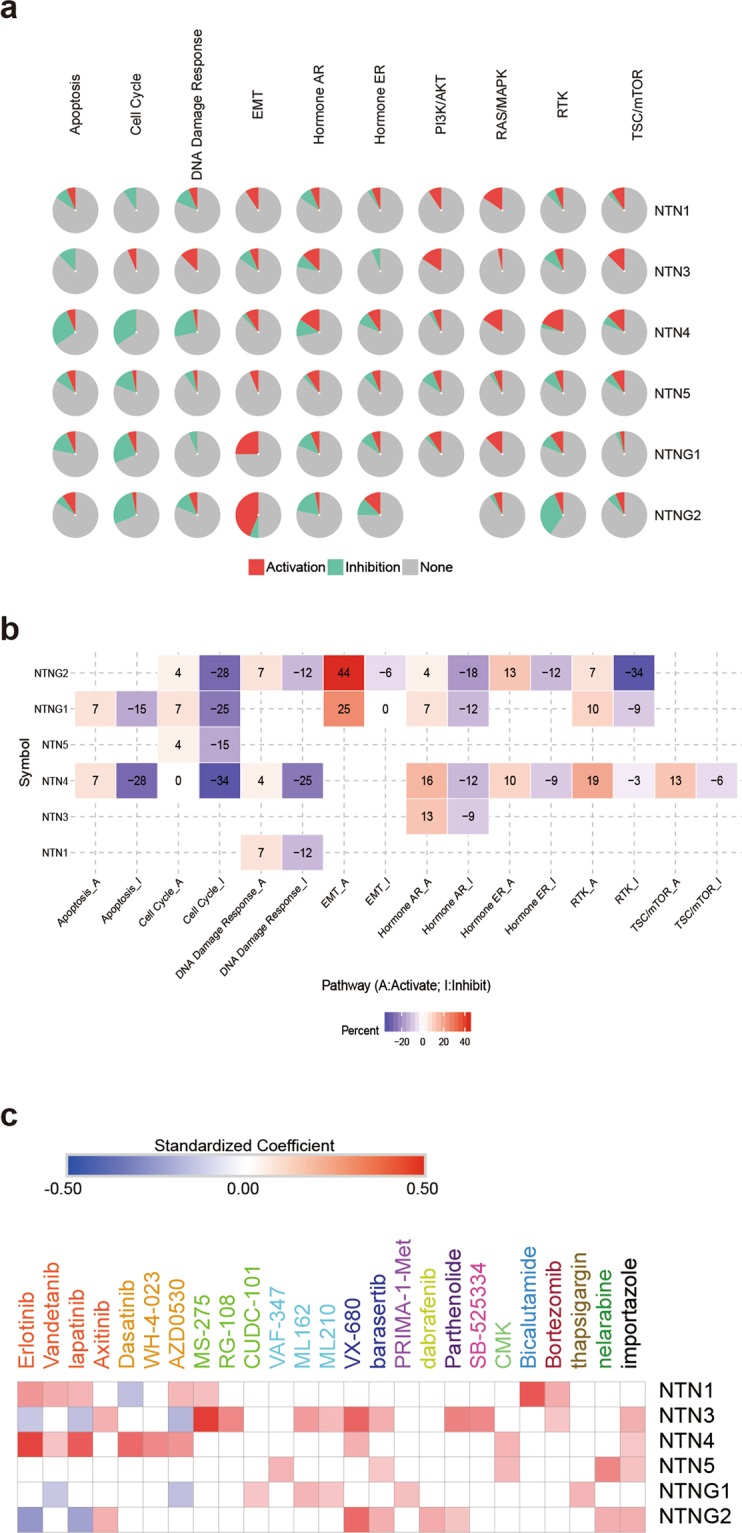
Netrins in pathways and sensitivity to drugs. (**a**) Global percentage shows percentage of gene’s function (activation or inhibition) for each pathway in all cancers. (**b**) Heatmap show netrin family members that have function (inhibit or activate) in at least 5 cancer types. Pathway A represent activation of this pathway, inhibition in a similar way showed as pathway I. (**c**) Heatmap of drug sensitivity / tolerance of netrins with high expression.

We analyzed the role of netrin family members in cancer pathways by GSCALite (Fig. 9a,b). NTN4 exhibited the strongest inhibition of apoptosis, cell cycle, and DNA damage response in the netrin family, showing a complete inhibitory effect on cell cycle. NTNG2 had the strongest activation of EMT, and the strongest inhibition of hormones ER and RTK. Overall, NTN4, NTN5, NTNG1, and NTNG2 exhibited strong inhibition of cell cycle, and NTNG1 and NTNG2 showed strong activation of EMT.

We downloaded the list of cancer drugs related to the netrins through the cancer pharmacogenomics research database PharmacoDB^49^, selecting p < 0.05, standardized coefficient >0.1, and the five most sensitive cancer drugs (Fig. 9c, Supplementary Data 11). SRC is an important kinase that mediates netrin signals. Highly expressed NTN4 is more sensitive to SRC inhibitors such as Dasatinib, WH-4-023, and AZD0530. Axitinib is a novel multi-target inhibitor for Abl, Src, and c-Kit to treat chronic myeloid leukemia. Highly expressed NTN3 and NTNG2 are more sensitive to Axitinib. These results strongly suggest that rational use of Src inhibitors is effective in treating tumors with high expression of certain netrin family members. Erlotinib, Vandetanib, Lapatinib, and Axitinib are FDA-approved drugs that affect protein tyrosine kinases, inhibit EGFR and VEGFR, and are used to treat cancers such as lung cancer, breast cancer, thyroid cancer, and melanoma^50^. The highly expressed NTN1 and NTN4 are sensitive to Erlotinib and Lapatinib. This suggests that the signaling pathway of NTN1 and NTN4 may be closely related to the signaling pathway mediated by EGF and VEGF, but it also indicates that the rational use of inhibitors of EGF/VEGF signaling pathway can effectively treat some tumors with high expression of Netrin family members. The high expression of NTN1, NTN3, and NTNG1 is also sensitive to MS-275 (HDAC inhibitor), RG-108 (DNA methyltransferase inhibitor), and CUDC-101 (HDAC inhibitor, EGFR inhibitor, and HER2 inhibitor), which inhibit tumor by affecting epigenesis. We speculate that epigenetic interactions of NTN1, NTN3, and NTNG1 may also be affected by these drugs. VAF-347, ML162, and ML210 are drugs that control cancer by altering cancer metabolism. Highly expressed NTN3, NTN5, and NTNG1 are correlated and sensitive to these three drugs, suggesting that the netrin family may be involved in tumor metabolism. The high mesenchymal state observed in human tumors and cancer cell lines is associated with resistance to multiple treatments of various cancer lineages. In particular, ML162 and ML210 are compounds that target and change the mesenchymal state, inhibit GPX4 activity, and promote apoptosis^51^. Because high expression of NTN3 and NTNG1 is sensitive to these compounds, we can speculate that NTN3 and NTNG1 may be involved in the mesenchymal state of cancer. In neuroblastoma and neuroendocrine prostate cancer, Aurora A exerts carcinogenic effects through interaction with N-Myc^52,53^, while VX-680 and Barasertib affect the cell cycle by inhibiting Aurora Kinase. Highly expressed NTN3 and NTNG2 cells are sensitive to these drugs, suggesting NTN3 and NTNG2 may be involved in N-Myc-related pathways and affect androgen-independent prostate cancer development. Bicalutamide is an FDA-approved drug that treats prostate cancer by binding to androgen to compete with androgen receptors^54^. Highly expressed NTN1 and NTN3 are both sensitive to Bicalutamide. We speculate that NTN1 and NTN3 may be potential targets for hormone-sensitive prostate cancer.

## Discussion

It is important to study netrin expression and regulation patterns in different cancers for the diagnosis and treatment of tumors with abnormal netrin expression. The focus of this study was to elucidate the comprehensive molecular characteristics and potential clinical significance of the netrin family in different types of cancer. Our findings are as follows: (1) members of the Netrin family are tightly regulated by multiple mechanisms at the genetic, transcriptional, and post-transcriptional levels; (2) mutations of members of the Netrin family correlate with tumor genetic characteristics; (3) Netrins may play important roles in the occurrence and development of endocrine system-related tumors and sex hormone targeting tumors; (4) Netrin family members may be promising prognostic indicators and potential therapeutic targets for lung and kidney cancer; (5) NTNG1 and NTNG2 are potential diagnostic markers and therapeutic targets that should be further studied systematically. To the best of our knowledge, this is the first report based on data mining and deep biological information analysis on the comprehensive molecular characteristics of the netrin family in pan-cancer. Our results demonstrate the utility of applying computational biology methods in combination with experimental biology to discover and elucidate new molecular biology mechanisms of cancer in the netrin family.

This study was the first comprehensive analysis of tumor genetic characteristics of mutations in members of the Netrin family. We found that tumor mutations of members of the Netrin family showed a unique distribution pattern for cancer type, protein structure, and ethnic group. It is worth noting that a significant number of missense or truncating mutations have been found in the Laminin N-terminal and EGF domains that interact with related receptors^55,56^ and the regulatory localization of the NTR domain^57,58^. Thus, the molecular mechanisms related to receptor affinity and spatial localization should be thoroughly investigated at the level of structural biology and biochemistry.

This study utilized multidimensional data mining, which revealed the potential value of Netrin family members in the diagnosis and treatment of specific cancers. To date, roles for only NTN1 and NTN4 have been described in breast cancer, ovarian cancer, prostate cancer, and pancreatic cancer among endocrine system-related tumors and sex hormone-targeting tumors. Our mutation analysis found highly enriched mutation of netrin family genes in uterine corpus endometrial carcinoma. From the methylation/expression-clinical analysis model, we further found association of abnormal expression patterns of some members of the netrin family with the cancer biological behavior of endocrine system-related tumors or sex hormone targeting tumors. NTN1, for example, is associated with UCEC’s MSI phenotype. More importantly, based on the comprehensive analysis of transcription, epigenetics, and signaling pathways, we found that in endocrine system-related tumors or sex hormones targeting organ tumors, the netrins may be regulated by a variety of transcription factors or chromatin modification factors known to be involved in regulation of cancer and related signaling pathways. For example, NTN3 is regulated by AR and EZH2 in prostate cancer, and also acts in the activation of the hormone ER pathway. Bicalutamide is an FDA approved drug for the treatment of prostate cancer, and is also sensitive to NTN3. Potential fusion genes and tumor-specific eQTL analysis also strongly suggest that netrins play important regulatory roles in the occurrence and development of some endocrine system-related tumors or sex hormone-producing organ tumors. However, only NTN1 was previously associated with the occurrence and development of kidney cancer and non-small cell lung cancer^59–61^. This study found that the NTN family not only has a high overall mutation rate in lung cancer, but that netrin activity is also closely related to the survival and clinical parameters of kidney cancer and non-small cell lung cancer. The MKL1__NTNG1 predicted by fusion gene prediction algorithm may also play a potentially important role in the development of lung adenocarcinoma. The association analysis of expression-eQTL-GWAS also revealed that the regulatory sites of netrins are related to kidney cancer and non-small cell lung cancer. More importantly, the sensitivity of highly expressed netrin family members to FDA-approved drugs for lung cancer is inconsistent and suggests an involvement in the mechanism of tumor resistance. These findings strongly suggest the clinical value of evaluating Netrin family members in making specific cancer diagnosis and treatment decisions.

An unexpected finding in this study was the identification of the potential cancer biological significance of NTNG1 and NTNG2. NTNG1 and NTNG2 show complementary expression in brain tissue^62^ and regulate the synaptic function of neural circuits in different ways^63^. NTNG1 and NTNG2 are associated with schizophrenia and bidirectional affective disorder^64–67^. Here we find that the expression or methylation of NTNG1 and NTNG2 is associated with survival and other clinical parameters of more than 10 types of cancer. There are also important epigenetic and transcriptional modifications of netrins that occur in pan-cancer that are related to the activation of the EMT pathway. More importantly, high expression of NTNG1 and NTNG2 is highly sensitive to some drugs. It is worth noting that the mutations and potential mirRNA targeting of NTNG1 and NTNG2 in cancer exhibit rates that are much higher than those predicted for NTN1 and NTN4. TCGA fusion transcripts analysis suggests the presence of fusion transcripts composed of NTNG1 or NTNG2 in multiple cancers. These findings strongly suggest that NTNG1 and NTNG2 may be potential tumor diagnostic markers and may be reasonable therapeutic targets worthy of future study.

It is worth noting that although members of the Netrin family generally exhibit characteristics of oncogenes, they also show down-regulated expression in some tumors. One possible explanation for this variation is that in many human cancers, up-regulation of Netrin family members inhibits apoptosis that is induced by dependent receptors such as DCC and UNC5H, thus promoting tumor progression. However, in some cancers, the selective inhibition of this receptor-dependent apoptosis pathway depends on the silencing of pro-apoptotic proteins. For example, a considerable number of human breast tumors and lung tumors show concurrent down-regulated expression of DNA methylation-dependent NTN1 and DAPK1. DAPK1 is a serine threonine kinase responsible for UNC5H-induced apoptosis. Inhibition of DNA methylation by drugs such as dexitabine can restore the expression of NTN1 and DAPK1 in cancer cells with low expression of Netrin-1^37,38,68^. Therefore, the combination of DNA methylation inhibitors and netrin-1 neutralizers may be an effective anticancer strategy. Further experimental studies are needed to explore the feasibility and universality of this combined therapy.

Overall, this study provides a comprehensive description of the molecular characteristics of the netrin family based on multidimensional data of genetics, epigenetics, and pharmacogenomics. We not only reveal the important role of the netrin family in the development of endocrine-related tumors and sex hormones targeting organ tumors, but suggest that NTNG1 and NTNG2 may be potential tumor diagnostic markers and treatment targets that warrant future in-depth systematic research. Additional experimental work is required to further analyze and validate these findings.

## Methods

### Mutation analysis

Mutual Exclusivity of netrins family members in pan-cancer was analyzed using Cbioportal (http://www.cbioportal.org/). TCGA mutation data of netrins were downloaded from TCGAbiolinksGUI^69^ on October 24, 2018 related to ACC (Adrenocortical carcinoma), BLCA (Bladder Urothelial Carcinoma), BRCA (Breast invasive carcinoma), CESC (Cervical squamous cell carcinoma and endocervical adenocarcinoma), CHOL (Cholangiocarcinoma), COAD (Colon adenocarcinoma), DLBC (Lymphoid Neoplasm Diffuse Large B-cell Lymphoma), ESCA (Esophageal carcinoma), GBM (Glioblastoma multiforme), HNSC (Head and Neck squamous cell carcinoma), KICH (Kidney Chromophobe), KIRC (Kidney renal clear cell carcinoma), KIRP (Kidney renal papillary cell carcinoma), LAML (Acute Myeloid Leukemia), LGG (Brain Lower Grade Glioma), LIHC (Liver hepatocellular carcinoma), LUAD (Lung adenocarcinoma), LUSC (Lung squamous cell carcinoma), MESO (Mesothelioma), OV (Ovarian serous cystadenocarcinoma), PAAD (Pancreatic adenocarcinoma), PCPG (Pheochromocytoma and Paraganglioma), PRAD (Prostate adenocarcinoma), READ (Rectum adenocarcinoma), SARC (Sarcoma), SKCM (Skin Cutaneous Melanoma), STAD (Stomach adenocarcinoma), TGCT (Testicular Germ Cell Tumors), THCA (Thyroid carcinoma), THYM (Thymoma), UCEC (Uterine Corpus Endometrial Carcinoma), UCS (Uterine Carcinosarcoma), and UVM (Uveal Melanoma). We then removed silent, 3′UTR, 5′Flank, Splice Region, and other mutations in order to study only the non-synonymous mutations in the coding region of the gene. We then classified Frame Shift Del, Nonsense Mutation, Frame Shift Del, Frame Shift Ins, and Splice Site as truncating mutations. TCGA patients were divided into four genetic ancestry groups (AA-African American, NA-Native American, EAA-East Asian American, and EA-European American) by TCGAA (http://52.25.87.215/TCGAA/). The optimal VEST3 and REVEL algorithms in VarCards^70^ (http://varcards.biols.ac.cn/) were used to screen for harmful mutations of the above patients with complete information (Supplementary Data 1). We used GraphPad Prism 7^71^ for statistical analysis. We downloaded data describing the functional and structural importance of protein sequences of netrins from UET (http://mammoth.bcm.tmc.edu/uet/) on Nov. 13, 2018, and selected coverage <0.1 as the parameter (Supplementary Data 12). Mutation information for netrins was downloaded from CCLE (https://portals.broadinstitute.org/ccle/about) on Nov. 18, 2018. The mutation sites were mapped using OncoPrinter (http://www.cbioportal.org/oncoprinter) and Mutation Mapper (http://www.cbioportal.org/ mutation_mapper).

### Fusion gene analysis

The fusion gene data of netrin family members were downloaded from the TCGA Fusion Gene Database (http://www.tumorfusions.org/) on December 9, 2018 (Supplementary Data 2). The database includes fusion genes predicted by PRADA analysis of RNA sequencing data of 33 kinds of TCGA cancers. Tier 1 and tier 2 are high confidence.

### Expression and clinical analysis

On December 11, 2018, the median expression value and survival curve P value of netrin family members in 33 kinds of TCGA cancer and adjacent tissues were downloaded from GEPIA (http://gepia.cancer-pku.cn/index.html) (Supplementary Data 3). On December 12, 2018, we downloaded the optimal survival curve P values for Bladder Carcinoma (BLCA), Breast cancer (BRCA), Cervical squamous cell carcinoma (CESC), Esophageal carcinoma (ESCA), Head-neck squamous cell carcinoma (HNSC), Kidney renal clear cell carcinoma (KIRC), Kidney renal papillary cell carcinoma (KIRP), Liver hepatocellular carcinoma (LIHC), Lung adenocarcinoma (LUAD), Lung squamous cell carcinoma (LUSC), Ovarian cancer (OV), Pancreatic ductal adenocarcinoma (PAAD), Pheochromocytoma and Paraganglioma (PCPG), Rectum adenocarcinoma (READ), Sarcoma (SARC), Stomach adenocarcinoma (STAD), Testicular Germ Cell Tumor (TGCT), Thymoma (THYM), Thyroid carcinoma (THCA), and Uterine corpus endometrial carcinoma (UCEC) from Kaplan-Meier Plotter (http://kmplot.com/analysis/). These data were merged with data from GEPIA (Supplementary Data 4). On November 19, 2018, we downloaded data to determine the relationship between the expression of netrin family members in 32 types of TCGA cancer and clinical parameters from LinkedOmics (http://www.linkedomics.org/login.php) (Supplementary Data 5). The expression heat map of netrin mRNA was downloaded from exoRBase (http://www.exoRBase.org).

### Methylation and clinical analysis

We downloaded the bubble map showing differential methylation of the netrins and downstream genes between 33 TCGA cancers and corresponding normal tissues. The correlation between methylation and expression of netrins and downstream genes in TCGA cancers and the correlation of differential survival with high and low methylation of netrins were determined for TCGA cancers using data obtained from GSCALite (http://bioinfo.life.hust.edu.cn/web/GSCALite/). On November 19, 2018, we downloaded data of the relationships between the methylation of netrin family members in 32 types of TCGA cancer and clinical parameters from LinkedOmics (http://www.linkedomics.org/login.php) (Supplementary Data 5).

### Transcription and epigenetics analysis

On November 16, 2018, we downloaded data from CHIPBASE V2.0 (http://rna.sysu.edu.cn/chipbase/) (Supplementary Data 7) for transcription factors and chromatin remodeling factors with binding sites positioned within 1 kb upstream or downstream of netrin family members. The co-expression between each factor and the netrin family member was evaluated in 32 kinds of TCGA cancer. We considered a correlation with an absolute value greater than 0.2, p < 0.05 as a statistically significant cut off. We downloaded mirRNA data and the location of potential binding sites on the 3′UTR of netrin family members from miRWalk (http://mirwalk.umm.uni-heidelberg.de/) (Supplementary Data 8) on December 10, 2018. The experimentally based mirRNAs that bind to the NTN family were queried from STARBASE v3.0 (http://starbase.sysu.edu.cn/) (Supplementary Data 9). After comparing the mirRNAs predicted in miRWalk and STARBASE v3.0, 42 common mirRNAs were selected. In STARBASE v3.0, the correlation between the expression in pan-cancer and the expression of the netrin family members was investigated. With FDR < 0.05 and negative correlation as screening conditions, 32 mirRNAs were finally selected.

### Pancan-eQTL analysis

On November 18, 2018, the cis-eQTLs for netrin family members in 33 cancers of TCGA were downloaded from the PancanQTL (http://bioinfo.life.hust.edu.cn/PancanQTL/) (Supplementary Data 10). The NTN1-related eQTLs were imported into Regulomedb (http://www.regulomedb.org/index) to determine whether each eQTL affects transcriptional regulation.

### Drug and pathway analysis

The global percentage map and the heatmap percentage map of the netrin family member genes in 10 cancer-related pathways were downloaded from GSCALite (http://bioinfo.life.hust.edu.cn/web/GSCALite/). All data for netrin family-related drugs were downloaded from PharmacoDB (https://pharmacodb.pmgenomics.ca/) on November 27, 2018, and an absolute value of correlation greater than 0.1, p < 0.05 was used as a meaningful cut off (Supplementary Data 11).

### Statistical analysis

We performed all heat map analysis and visualization in Morpheus (https://software.broadinstitute.org/morpheus/). Online analysis websites and software of omicshare (http://www.omicshare.com/tools/), BDP (https://me.bdp.cn/home.html), Cytoscape (v.3.6.1), and IBS (1.0.3) were also used. The TCGAbiolinksGUI package was used in the RStudio programming environment (v1.1.456).

## Supplementary information

Dataset 1.

Dataset 2.

Dataset 3.

Dataset 4.

Dataset 5.

Dataset 6.

Dataset 7.

Dataset 8.

Dataset 9.

Dataset 10.

Dataset 11.

Dataset 12.

## Data Availability

All data generated or analysed during this study are included in this published article (and its Supplementary Information Files).

## References

[1] Patel S, Ngounou WA, Darie CC, Clarkson BD (2014). Cancer secretomes and their place in supplementing other hallmarks of cancer. Adv Exp Med Biol.

[2] Greening DW, Gopal SK, Xu R, Simpson RJ, Chen W (2015). Exosomes and their roles in immune regulation and cancer. Semin Cell Dev Biol.

[3] Lasser C (2016). Exosomes in the nose induce immune cell trafficking and harbour an altered protein cargo in chronic airway inflammation. J Transl Med.

[4] Record M, Poirot M, Silvente-Poirot S (2014). Emerging concepts on the role of exosomes in lipid metabolic diseases. Biochimie.

[5] Salem KZ (2016). Exosomes in Tumor Angiogenesis. Methods Mol Biol.

[6] Yao L, Zhang Y, Chen K, Hu X, Xu LX (2011). Discovery of IL-18 as a novel secreted protein contributing to doxorubicin resistance by comparative secretome analysis of MCF-7 and MCF-7/Dox. Plos One.

[7] Takata T (2012). Characterization of proteins secreted by pancreatic cancer cells with anticancer drug treatment *in vitro*. Oncol Rep.

[8] Kim R, Emi M, Tanabe K (2006). Cancer immunosuppression and autoimmune disease: beyond immunosuppressive networks for tumour immunity. Immunology.

[9] Jakobsen KR (2015). Exosomal proteins as potential diagnostic markers in advanced non-small cell lung carcinoma. J Extracell Vesicles.

[10] Logozzi M (2009). High levels of exosomes expressing CD63 and caveolin-1 in plasma of melanoma patients. Plos One.

[11] Straussman R (2012). Tumour micro-environment elicits innate resistance to RAF inhibitors through HGF secretion. Nature.

[12] Hedgecock EM, Culotti JG, Hall DH (1990). The unc-5, unc-6, and unc-40 genes guide circumferential migrations of pioneer axons and mesodermal cells on the epidermis in C. elegans. Neuron.

[13] Feinstein J, Ramkhelawon B (2017). Netrins & Semaphorins: Novel regulators of the immune response. Biochim Biophys Acta Mol Basis Dis.

[14] Ylivinkka I, Keski-Oja J, Hyytiainen M (2016). Netrin-1: A regulator of cancer cell motility?. Eur J Cell Biol.

[15] Gamage DG, Hendrickson TL (2013). GPI transamidase and GPI anchored proteins: oncogenes and biomarkers for cancer. Crit Rev Biochem Mol Biol.

[16] Mehlen P, Tauszig-Delamasure S (2014). Dependence receptors and colorectal cancer. Gut.

[17] Mehlen P, Mazelin L (2003). The dependence receptors DCC and UNC5H as a link between neuronal guidance and survival. Biol Cell.

[18] Castets M, Mehlen P (2010). Netrin-1 role in angiogenesis: to be or not to be a pro-angiogenic factor?. Cell Cycle.

[19] Shimizu A (2013). Netrin-1 promotes glioblastoma cell invasiveness and angiogenesis by multiple pathways including activation of RhoA, cathepsin B, and cAMP-response element-binding protein. J Biol Chem.

[20] Ylivinkka I (2013). Netrin-1-induced activation of Notch signaling mediates glioblastoma cell invasion. J Cell Sci.

[21] Ko SY, Blatch GL, Dass CR (2014). Netrin-1 as a potential target for metastatic cancer: focus on colorectal cancer. Cancer Metastasis Rev.

[22] Hu Y (2012). Netrin-4 promotes glioblastoma cell proliferation through integrin beta4 signaling. Neoplasia.

[23] Eveno C (2011). Netrin-4 delays colorectal cancer carcinomatosis by inhibiting tumor angiogenesis. Am J Pathol.

[24] An XZ (2016). Netrin-1 suppresses the MEK/ERK pathway and ITGB4 in pancreatic cancer. Oncotarget.

[25] Gao J (2013). Integrative analysis of complex cancer genomics and clinical profiles using the cBioPortal. Sci Signal.

[26] Cerami E (2012). The cBio cancer genomics portal: an open platform for exploring multidimensional cancer genomics data. Cancer Discov.

[27] Hu X (2018). TumorFusions: an integrative resource for cancer-associated transcript fusions. Nucleic Acids Res.

[28] Ma Z (2001). Fusion of two novel genes, RBM15 and MKL1, in the t(1;22)(p13;q13) of acute megakaryoblastic leukemia. Nat Genet.

[29] Cheng X (2015). MKL1 potentiates lung cancer cell migration and invasion by epigenetically activating MMP9 transcription. Oncogene.

[30] Tang Z (2017). GEPIA: a web server for cancer and normal gene expression profiling and interactive analyses. Nucleic Acids Res.

[31] Lanczky A (2016). miRpower: a web-tool to validate survival-associated miRNAs utilizing expression data from 2178 breast cancer patients. Breast Cancer Res Treat.

[32] Li, S. *et al*. exoRBase: a database of circRNA, lncRNA and mRNA in human blood exosomes. *Nucleic Acids Res* **46**, D106-D112 (2018). 10.1093/nar/gkx891 PMC5753357 30053265

[33] Hurwitz SN (2016). Proteomic profiling of NCI-60 extracellular vesicles uncovers common protein cargo and cancer type-specific biomarkers. Oncotarget.

[34] Wang Z, Hill S, Luther JM, Hachey DL, Schey KL (2012). Proteomic analysis of urine exosomes by multidimensional protein identification technology (MudPIT). Proteomics.

[35] Hong BS (2009). Colorectal cancer cell-derived microvesicles are enriched in cell cycle-related mRNAs that promote proliferation of endothelial cells. Bmc Genomics.

[36] Liu CJ (2018). GSCALite: a web server for gene set cancer analysis. Bioinformatics.

[37] Grandin M (2016). Structural Decoding of the Netrin-1/UNC5 Interaction and its Therapeutical Implications in Cancers. Cancer Cell.

[38] Grandin M (2016). Inhibition of DNA methylation promotes breast tumor sensitivity to netrin-1 interference. Embo Mol Med.

[39] Vasaikar SV, Straub P, Wang J, Zhang B (2018). LinkedOmics: analyzing multi-omics data within and across 32 cancer types. Nucleic Acids Res.

[40] Zhou KR (2017). ChIPBase v2.0: decoding transcriptional regulatory networks of non-coding RNAs and protein-coding genes from ChIP-seq data. Nucleic Acids Res.

[41] Zheng N (2018). Rottlerin inhibits cell growth and invasion via down-regulation of EZH2 in prostate cancer. Cell Cycle.

[42] Yan, K.S. *et al*. EZH2 in Cancer Progression and Potential Application in Cancer Therapy: A Friend or Foe? *Int J Mol Sci* **18** (2017). 10.3390/ijms18061172 PMC5485996 28561778

[43] Kim BS (2018). An immunohistochemical panel consisting of EZH2, C-KIT, and CD205 is useful for distinguishing thymic squamous cell carcinoma from type B3 thymoma. Pathol Res Pract.

[44] Kim GC (2018). Upregulation of Ets1 expression by NFATc2 and NFKB1/RELA promotes breast cancer cell invasiveness. Oncogenesis.

[45] Han B (2015). Metformin inhibits thyroid cancer cell growth, migration, and EMT through the mTOR pathway. Tumour Biol.

[46] Sticht C, De La Torre C, Parveen A, Gretz N (2018). miRWalk: An online resource for prediction of microRNA binding sites. Plos One.

[47] Li JH, Liu S, Zhou H, Qu LH, Yang JH (2014). starBase v2.0: decoding miRNA-ceRNA, miRNA-ncRNA and protein-RNA interaction networks from large-scale CLIP-Seq data. Nucleic Acids Res.

[48] Gong J (2018). PancanQTL: systematic identification of cis-eQTLs and trans-eQTLs in 33 cancer types. Nucleic Acids Res.

[49] Smirnov P (2018). PharmacoDB: an integrative database for mining *in vitro* anticancer drug screening studies. Nucleic Acids Res.

[50] Lee, C.S., Baek, J. & Han, S.Y. The Role of Kinase Modulators in Cellular Senescence for Use in Cancer Treatment. *Molecules* **22** (2017). 10.3390/molecules22091411 PMC6151769 28841181

[51] Viswanathan VS (2017). Dependency of a therapy-resistant state of cancer cells on a lipid peroxidase pathway. Nature.

[52] Otto T (2009). Stabilization of N-Myc is a critical function of Aurora A in human neuroblastoma. Cancer Cell.

[53] Mosquera JM (2013). Concurrent AURKA and MYCN gene amplifications are harbingers of lethal treatment-related neuroendocrine prostate cancer. Neoplasia.

[54] Liu C (2017). Niclosamide and Bicalutamide Combination Treatment Overcomes Enzalutamide- and Bicalutamide-Resistant Prostate Cancer. Mol Cancer Ther.

[55] Boyer NP, Gupton SL (2018). Revisiting Netrin-1: One Who Guides (Axons). Front. Cell. Neurosci..

[56] Meijers R, Smock RG, Zhang Y, Wang JH (2020). Netrin Synergizes Signaling and Adhesion through DCC. Trends Biochem. Sci..

[57] Meneret A (2017). Mutations in the Netrin-1 Gene Cause Congenital Mirror Movements. J. Clin. Invest..

[58] Delloye-Bourgeois C (2012). Nucleolar Localization of a Netrin-1 Isoform Enhances Tumor Cell Proliferation. Sci. Signal..

[59] Yildirim ME, Kefeli U, Aydin D, Sener N, Gumus M (2016). The value of plasma netrin-1 in non-small cell lung cancer patients as diagnostic and prognostic biomarker. Tumour Biol.

[60] Zhang X (2018). Netrin-1 elicits metastatic potential of non-small cell lung carcinoma cell by enhancing cell invasion, migration and vasculogenic mimicry via EMT induction. Cancer Gene Ther.

[61] Zhan B, Kong C, Guo K, Zhang Z (2013). PKCalpha is involved in the progression of kidney carcinoma through regulating netrin-1/UNC5B signaling pathway. Tumour Biol.

[62] Nakashiba T, Nishimura S, Ikeda T, Itohara S (2002). Complementary expression and neurite outgrowth activity of netrin-G subfamily members. Mech Dev.

[63] Zhang Q (2016). Diversification of behavior and postsynaptic properties by netrin-G presynaptic adhesion family proteins. Mol Brain.

[64] Faraone SV, Lasky-Su J, Glatt SJ, Van Eerdewegh P, Tsuang MT (2006). Early onset bipolar disorder: possible linkage to chromosome 9q34. Bipolar Disord.

[65] Venken T (2005). Genomewide scan for affective disorder susceptibility Loci in families of a northern Swedish isolated population. Am J Hum Genet.

[66] Cheng R (2006). Genome-wide linkage scan in a large bipolar disorder sample from the National Institute of Mental Health genetics initiative suggests putative loci for bipolar disorder, psychosis, suicide, and panic disorder. Mol Psychiatry.

[67] Eastwood SL, Harrison PJ (2008). Decreased mRNA expression of netrin-G1 and netrin-G2 in the temporal lobe in schizophrenia and bipolar disorder. Neuropsychopharmacol.

[68] Tang X (2004). Hypermethylation of the Death-Associated Protein Kinase Promoter Attenuates the Sensitivity to TRAIL-induced Apoptosis in Human Non-Small Cell Lung Cancer Cells. Mol. Cancer Res..

[69] Colaprico A (2016). TCGAbiolinks: an R/Bioconductor package for integrative analysis of TCGA data. Nucleic Acids Res.

[70] Li J (2018). VarCards: an integrated genetic and clinical database for coding variants in the human genome. Nucleic Acids Res.

[71] Mitteer DR, Greer BD, Fisher WW, Cohrs VL (2018). Teaching behavior technicians to create publication-quality, single-case design graphs in graphpad prism 7. J Appl Behav Anal.

